# Is Palatal Rugae Pattern a Reliable Tool for Personal Identification following Orthodontic Treatment? A Systematic Review and Meta-Analysis

**DOI:** 10.3390/diagnostics12020418

**Published:** 2022-02-06

**Authors:** Archana A. Gupta, Supriya Kheur, Abdulrahman Alshehri, Wael Awadh, Zeeshan Heera Ahmed, Shaikh Mohammed Abdul Feroz, Samar Saeed Khan, Shazia Mushtaq, Harisha Dewan, Zohaib Khurshid, Saranya Varadarajan, Govindarajan Sujatha, Vishnu Priya Veeraraghavan, Shankargouda Patil

**Affiliations:** 1 Private Dental Clinic, Pune 411033, India; archanaanshumangupta@gmail.com; 2 Department of Oral Pathology and Microbiology, Dr. D. Y. Patil Dental College and Hospital, Dr. D. Y. Patil Vidyapeeth, Pune 411018, India; supriya.kheur@dpu.edu.in; 3 Division of Orthodontics, Department of Preventive Dental Sciences, College of Dentistry, Jazan University, Jazan 45142, Saudi Arabia; aalshehri@jazanu.edu.sa (A.A.); awadhwael@gmail.com (W.A.); 4 Department of Restorative Dental Sciences, College of Dentistry, King Saud University, Riyadh 11451, Saudi Arabia; aheera@ksu.edu.sa; 5 Department of Prosthetic Dental Science, College of Dentistry, Jazan University, Jazan 45142, Saudi Arabia; feroz77777@gmail.com (S.M.A.F.); harisha.dewan@yahoo.com (H.D.); 6 Department of Maxillofacial Surgery and Diagnostic Sciences, Division of Oral Pathology, College of Dentistry, Jazan University, Jazan 45142, Saudi Arabia; samarkhan8@gmail.com; 7 Dental Health Department, College of Applied Medical Sciences, King Saud University, Riyadh 11451, Saudi Arabia; smushtaqdr@gmail.com; 8 Department of Prosthodontics and Dental Implantology, College of Dentistry, King Faisal University, Al-Ahsa 31982, Saudi Arabia; drzohaibkhurshid@gmail.com; 9 Department of Oral Pathology and Microbiology, Sri Venkateswara Dental College and Hospital, Chennai 600130, India; vsaranya87@gmail.com (S.V.); gsuja@rediffmail.com (G.S.); 10 Saveetha Institute of Medical and Technical Sciences, Saveetha Dental College and Hospitals, Saveetha University, Chennai 600077, India; 11 Centre of Molecular Medicine and Diagnostics (COMManD), Department of Biochemistry, Saveetha Dental College and Hospitals, Saveetha Institute of Medical and Technical Sciences, Saveetha University, Chennai 600077, India; drvishnupriyav@gmail.com

**Keywords:** forensic odontology, morphology, orthodontic treatment, palatal rugae

## Abstract

Background: To qualitatively and quantitatively review the reliability of palatal rugae as a tool for personal identification following orthodontic treatment. Methods: Cross-sectional retrospective studies assessing the accuracy of matching palatal rugae pattern pre- and post-orthodontic treatment were identified from PubMed and SCOPUS databases. The title and abstract of the articles identified in the search were screened for potential duplicates and relevancy to the topic of interest. The full text of the articles selected in the screening was analyzed using the inclusion and exclusion criteria. Quantitative analysis of the studies representing coherent data in terms of age and treatment choice was performed using RevMan software. Results: Out of 64 screened articles, only 18 articles fulfilled the eligibility criteria and were included in the systematic review. Out of these 18 articles, only 3 studies had data compatible with the quantitative analysis. Significant changes were noted in lateral first rugae in transverse bilateral direction (*p* = 0.02) and between second and third lateral rugae of the left side in the anteroposterior direction (*p* = 0.04). Despite the dimensional changes, observers in most studies were able to accurately (>90%) match the palatal rugae pre- and post-orthodontic treatment through visual observation. Conclusion: The accuracy of the visual matching, despite the significant dimensional changes, indicates that morphology could have potentially been the major matching factor. Thus, a combination of dimensional and morphological evaluation of the palatal rugae could potentially increase the accuracy of personal identification.

## 1. Introduction

Palatal rugae are the irregular connective tissue also known as “plicae palatinae”. These are anatomical folds located posterior to the incisive papilla in the palatal anterior third. These folds, which form as early as 3 months in utero, are largely attributed to the hardened connective tissue that covers the bone. Their orientation and pattern are formed in the fourth month of intrauterine life. Their uniqueness lies in the fact that they are stable and show post-mortem resistance [1] until oral mucosal degeneration after death [2,3]. Their anatomical location on the internal aspect of the oral cavity protects them from different environmental factors including rising temperature, palatal infections, trauma, and tooth exfoliation and eruption [4,5,6]. They are considered suitable landmarks for forensic identification [7,8,9].

Palatine rugae analysis has been proved to be a good alternative or adjunct method for identification where teeth are lost. Allen proposed its potential role as a means for personal identification in 1889 and the term “Palatal rugoscopy” was first proposed by the Spanish investigator Trobo Hermosa in 1932 [10]. Palatal rugae can be recorded through casts or plaster models, which act as an essential aid in orthodontic diagnosis and treatment planning [9]. However, with evolving technology, diagnostic tools have evolved into digitized mapping and 3D scanning [11,12].

Though palatal rugae are still considered a reliable tool for identification, it is imperative to acknowledge rugae patterns can undergo changes due to several factors including orthodontic treatments, surgical palatal repairs, and extractions of adjacent teeth [13,14,15]. Questions have also been raised as to the growth-related stability of the rugae. Given the increasing literature suggesting the instability of palatal rugae, there appears to be a lack of consensus in identifications made solely based on the palatal rugae. Among the various factors suggested that cause changes in the palatal rugae, the effect of orthodontic treatment is the most well-explored. Thus, the present systematic review and meta-analysis were formulated to qualitatively and quantitatively analyze the effect of orthodontic treatment on the stability of palatine rugae.

## 2. Materials and Methods

Protocol and registration: PRISMA (Preferred Reporting Items for Systematic Reviews and Meta-Analyses) and MOOSE (Meta-Analysis of Observational Studies in Epidemiology) guidelines were strictly adhered to in the present systematic review and meta-analysis. The PROSPERO registration number for the review is CRD42020214804.

Inclusion criteria: Original studies in the English language discussing any changes in the palatal rugae pattern before and after orthodontic treatment were included.

Exclusion criteria: Reviews, short articles (commentary, letters, correspondent pieces), and articles not in the English language were excluded.

Focused question: “Are palatal rugae a reliable marker for personal identification following orthodontic treatment?”

Search strategy: Databases including the SCOPUS and PubMed were used for mining the data. Any relevant articles obtained from cross-referencing the screened articles were also included if they satisfied the inclusion criteria. Medical subject heading combinations, including Palatal rugae and forensic odontology, Palatal rugae and orthodontic treatment, Palatal rugae and orthodontic treatment, and forensic odontology were used for mining the data.

Study selection and data extraction: The review was conducted in two steps by two reviewers (S.S.K. and A.A.G.) as follows:

1st step: All the identified articles were screened using their title and abstract. Duplicates and articles irrelevant to the topic of interest were excluded.

2nd step: The articles included from the 1st step were analyzed using their full texts. Articles not satisfying the inclusion criteria were excluded. 

Vital data including the sample details, orthodontic treatment modality employed, and the mean changes noted in the palatal rugae were retrieved from the included studies.

Statistical analysis: Kappa statistics were used to assess the inter-observer reliability (Cohen’s kappa coefficient [κ]) between SK and AG. Quantitative analysis of the studies representing coherent data in terms of age and treatment choice has been performed using RevMan software, Version 5.4. Risk of bias analysis: Joanna Briggs’s critical appraisal tool for cross-sectional studies was employed to assess the risk of bias in the included studies. The reason for considering all the included studies as cross-sectional was that although the data of pre- and post-orthodontic treatment were extracted, they were extracted at a single time point as retrospective data. 

## 3. Results

**Figure 1:**
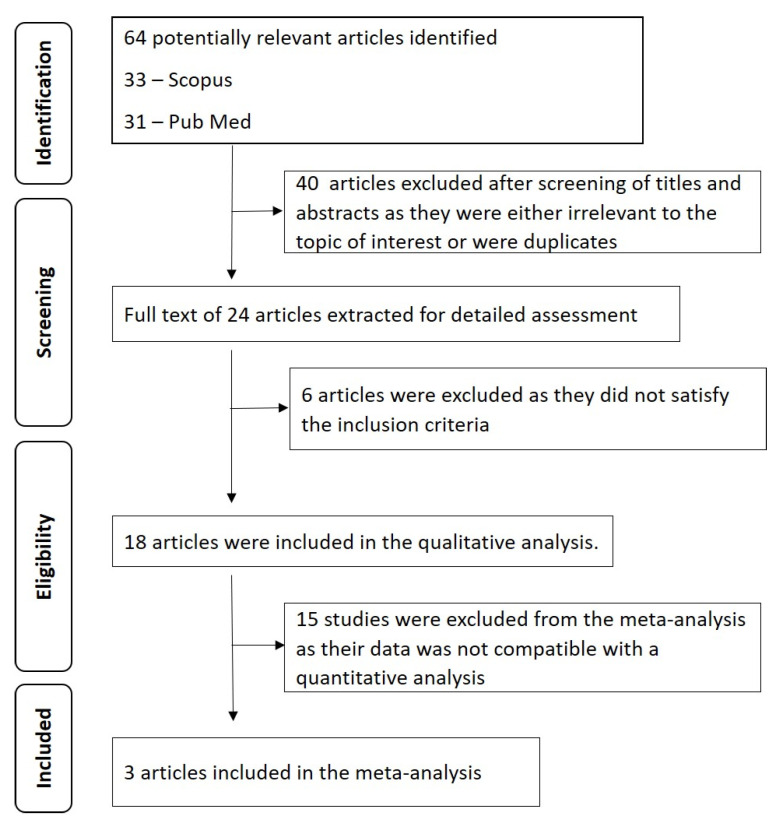
Prisma flowchart summarizing the workflow of the systematic review.

Study selection: The workflow of systematic review has been summarized in Figure 1. A total of 64 articles, including 31 from PubMed and 33 from Scopus, have been retrieved using the keywords. Screening the titles and abstracts of the identified articles revealed that 40 articles were either duplicate or were not related to the topic of interest and thus were excluded. The full text of the remaining 24 articles was assessed using the selection criteria. Only 18 fulfilled the eligibility criteria and were included in the systematic review. κ value of 0.96 and 0.98 was obtained for the 1st and 2nd step of the review process, indicating a good overall inter-observer reliability. Table 1 summarizes the data extracted from the included studies.

Joanna Brigg’s risk of bias assessment: Out of 18 studies, 11 showed a moderate risk of bias [8,19,20,22,24,26,27,29,30,31,32] and 7 showed a low risk of bias [16,17,18,21,23,25,28] (Table 2). Inclusion criteria have been properly defined by seven studies [17,18,21,23,25,28,32]. Details of the subjects included in the studies, objectives, and standard criteria used for the measurement and the reliability and validity of the exposure measurement tool have been described by thirteen studies [8,16,17,18,19,20,21,22,23,24,25,26,28]. Five studies did not provide details of the selected subjects [27,29,30,31,32]. Confounding factors were considered in three studies [16,23,25]. Reliability in outcome measurement and appropriate statistical analysis were assessed in all eighteen studies.

Study characteristics: Of the studies included in the review, six were from India [16,18,19,27,32], four from the United States of America [17,19,22,24], two from China [23,25], and one each from Japan [20], Egypt [21], Greece [26], Portugal [28], Jordan [30], and Pakistan [31]. All the included studies compared the palatal rugae pre- and post-orthodontic treatment. The orthodontic treatments used in the included studies were rapid maxillary expansion [18,32], retraction of maxillary anterior teeth with straight wire with or without extraction [8,19,20,21,22,23], use of headgears, and functional appliances including fixed orthodontic appliances [24,31] and pre-adjusted edgewise therapy [29].

Diagnostic Modalities used for comparing palatal rugae pre and post orthodontic treatment: Methods for evaluating the rugae included the use of plaster models or casts. Some authors also used 3D images of casts, scanning of the cast using a Simplex DP 30 computer scanner, and lateral cephalometric radiographs, while some studies included digital images of palatal rugae with DSLR [17,20,21,22,23]. Subjective assessment of casts was carried out by evaluating by visual matching by observers [19,23,25,26,28,29,30,32]. For objective evaluation, most of the studies used medial and lateral points of all the three rugae as landmarks and evaluated transverse and anteroposterior distances between them [8,17,18,19,20,21,24,30,32]. Other dimensional measurements in the included studies were: measurement of the perpendicular distances from the medial and lateral points until the mid-palatal raphae [21,24]; measurement of the inter-canine distance and evaluation of the ratio between inter-canine width and lateral dimension of the 3rd rugae [27]; evaluation of the molar and incisor movements relative to each ruga before and after treatment [22].

Qualitative analysis of the effect of orthodontic treatment on the palatal rugae: The studies gave conflicting results on the stability of the rugae pattern after orthodontic treatments. Shetty, D. et al., Xiu-Ping Wu et al., Jang, I. et al., Shukla, D. et al., Li, B. et al., Stavrianos, C. et al., Samyukta S, Abilasha R and Abdul Aziz, and H.M. et al. reported that the changes occurring with orthodontic treatment do not significantly alter the rugae patterns and hence rugae patterns can be used as reliable forensic markers [16,19,20,21,23,25,26,27]. Braga S and Caldas IM and Ali, B. et al. reported that only shapes (morphology) of palatal rugae can be considered a reliable marker for forensic identification but not the lengths (dimensions) [28,31]. Abdul Aziz, H.M. et al. and Shukla, D. et al. stated that the third palatal rugae are the most stable landmark to be used in forensic odontology [19,21]. Jang, I. et al. went one step further and reported the medial points of the third palatal rugae to be the most stable [20]. Bailey, L.J. et al. reported the usage of both medial and lateral points of the third palatal rugae as stable anatomic reference points [8]. Almeida, M.A. et al. questioned the stability of lateral points of all the three rugae and stressed the usage of medial points as stable reference points [24]. Kapoor P and Miglani R recorded the transverse changes in the rugae pattern in the order of 3rd rugae > 2nd rugae > 1st rugae [18]. Deepak, V. et al. and Mustafa, A. G. et al., however, questioned the reliability of these palatine rugae as forensic markers [29,30].

Quantitative analysis of the effect of orthodontic treatment on the palatal rugae: Out of the 18 studies in the systematic review, only 6 studies provided objective measurement in palatal rugae pattern (transverse or anteroposterior) concerning their medial and lateral points before and after orthodontic treatment [8,17,19,20,21,24]. The remaining 12 studies either presented only subjective data related to the morphological pattern of rugae or objective data (inter-canine width, distance from the molars and incisors) not related to transverse or anteroposterior measurements of the medial and lateral points of the rugae. In some studies, only visual matching by observers was used as the assessing tool. Also, 18 studies included in the systematic review differed in several parameters in their methodologies including the age of subjects selected, choice of treatment, duration of treatment, etc. Due to these methodological variations, only three studies with objective data could be included in the meta-analysis where the choice of treatment and age of the study participants were similar [8,19,21].

**Figure 2:**
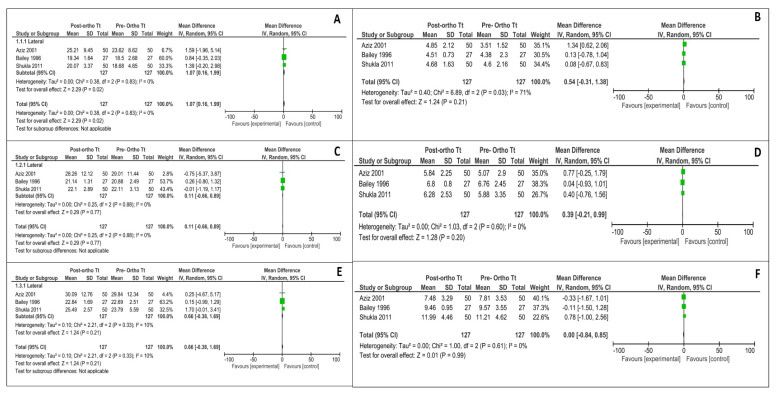
Meta-analysis summarizing the effect of the orthodontic treatment on the transverse dimensions. (**A**) Transverse bilateral changes in first palatal rugae (lateral); (**B**) Transverse bilateral changes in first palatal rugae (medial); (**C**) Transverse bilateral changes in second palatal rugae (lateral); (**D**) Transverse bilateral changes in second palatal rugae (medial); (**E**) Transverse bilateral changes in third palatal rugae (lateral); (**F**) Transverse bilateral changes in third palatal rugae (medial).

**Figure 3:**
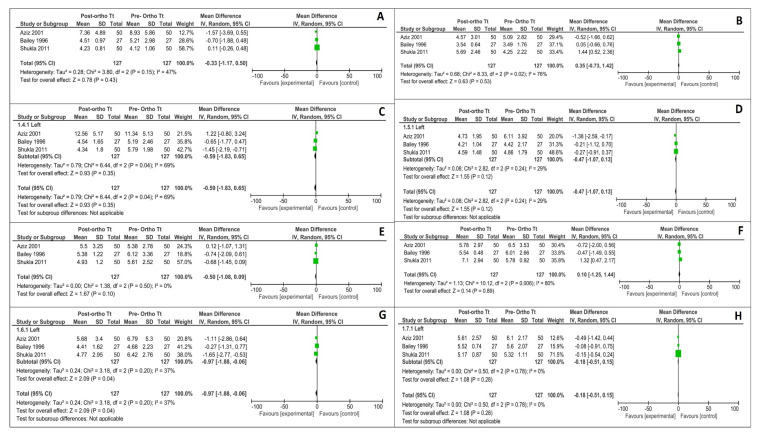
Meta-analysis summarizing the effect of the orthodontic treatment on the anteroposterior dimensions. (**A**) Antero-posterior changes between 1–2 rugae of right side (lateral); (**B**) Antero-posterior changes between 1–2 rugae of right side (medial); (**C**) Antero-posterior changes between 1–2 rugae of left side (lateral); (**D**) Antero-posterior changes between 1–2 rugae of left side (medial); (**E**) Antero-posterior changes between 2–3 rugae of right side (lateral); (**F**) Antero-posterior changes between 2–3 rugae of right side (medial); (**G**) Antero-posterior changes between 2–3 rugae of left side (lateral); (**H**) Antero-posterior changes between 2–3 rugae of the left side (medial).

Data from the three studies included in the meta-analysis was converted to mm (eg: 1 pixel = 0.264558 mm for an image of 96 dpi and 1 pixel = 0.08466 mm for an image of 300 dpi). The relevant data, including mean deviations before and after orthodontic treatment and respective standard deviations, were used to create a forest plot with the help of Revman software (Figure 2 and Figure 3). As depicted by the meta-analysis and the forest plots, significant changes have been observed in the lateral first rugae in transverse bilateral direction (*p* = 0.02) and two to three lateral rugae of the left side in the anteroposterior direction (*p* = 0.04). There were no significant changes noted in the rest of the assessed rugae dimensions.

## 4. Discussion

Palatal rugae are often used as an identification tool in forensic dentistry as the tissue is resistant to decomposition due to the protection rendered by the buccal pad of fat and teeth. Also, similar to the fingerprint, the palatal rugae pattern is unique for every individual and allows identification. The age changes in the palatal rugae pattern are minimal and hence could aid as an adjunct tool in forensic dentistry, especially in edentulous patients. However, significant changes in the rugae pattern could occur as a result of surgery, orthodontic treatment (which could range from minor corrections in the alignment of teeth to complicated procedures such as arch expansion, use of headgears, functional appliances, and orthognathic surgery), or extraction of teeth. Considerable published literature is available that has assessed and reported the palatal rugae pattern before and after orthodontic treatment. With the available information, the present systematic review with meta-analysis was formulated to assess the reliability of palatal rugae for personal identification following orthodontic treatment.

In the present systematic review, the nature of orthodontic treatment in the included articles ranged from rapid maxillary [18,32], retraction of maxillary anterior teeth with straight wire with or without extraction [8,19,20,21,22,23], use of headgears and functional appliances including fixed orthodontic appliances [24,31], and pre-adjusted edgewise therapy [29]. The diagnostic modalities used for the assessment of palatal rugae patterns varied among the included studies, leading to heterogeneity. However, Taneva, E.D. et al. had compared two different techniques for the assessment of the rugae pattern and reported no significant differences in the two techniques [17]. Hence it may be potentially inferred that the variations in the palatal rugae pattern reported by the included study due to the differences in the diagnostic modalities could be minimal.

Most of the included studies demonstrated no significant changes in morphologic alterations in the rugae pattern following orthodontic treatment and thus concluded that palatal rugae could be considered a reliable tool in forensic dentistry. Also, few studies had reported that the third palatal rugae were the most reliable landmark for forensic identification [7,8,18,20,21,24]. Considering the stability of medial and lateral points of the palatal rugae in rendering accurate forensic information, contradictory results were reported in the published data included in the present review. While Jang, I. et al. and Almeida, M.A. et al. reported the medial points of the third palatal rugae to be most stable, Bailey, L.J. et al. reported that both medial and lateral points of the third palatal rugae were stable anatomic reference points [8,20,24]. These differences could be attributed to the type of orthodontic treatment rendered to the study participants and the sample size employed in the studies. 

On the contrary, few studies have reported changes in the palatal rugae pattern following orthodontic treatment. Kapoor, P. and Miglani, R. reported that the 3rd rugae underwent maximum changes in the transverse pattern [18]. Deepak, V. et al. and Mustafa, A.G. et al. questioned the reliability of these palatine rugae as forensic markers [29,30]. This highlights the importance of maintaining dental records and cast models before and after orthodontic treatment, which could later render forensic information despite minor changes in the pattern. However, both the studies had a moderate risk of bias and did not report details of the selected study participants. The quantitative changes in the palatal rugae pattern before and after orthodontic treatment were assessed in only six out of the eighteen studies included. The remaining twelve studies had assessed only morphological alterations. However, only in three [8,19,21] out of six studies with quantitative data, the type of orthodontic treatment was similar and the age of the study participants was above 18 years. Age was a major factor as in participants below 18 years, the influence of the growth spurt on the rugae pattern could have been a potential confounder. A meta-analysis of the three studies revealed a significant transverse bilateral change in the lateral first rugae (*p* = 0.02) and anteroposterior change in the 2–3 lateral rugae of the left side (*p* = 0.04), respectively. There were no significant changes in the other rugae points assessed. The significant quantitative change in the transverse dimension of the lateral first rugae and the anteroposterior dimension of the second to third lateral rugae could be attributed to the fact that in all three studies, retraction of maxillary anterior teeth was carried out along with the extraction of premolars. Considering the role of tooth extraction on the palatal rugae pattern, very few studies had compared the changes in rugae pattern and reported no significant difference in the morphological pattern, while a few studies had only assessed the rugae pattern with orthodontic treatment and extraction [8,19,20,21,22,23]. However, in the present meta-analysis, we observed quantitative change in a transverse direction. Hence, the results must be viewed with caution owing to the heterogeneity of data and sample size. Further studies have to be carried out to assess the exact role of extraction on palatal rugae patterns [8,19,21].

Shukla, D. et al., Aziz, H.M.A., and Sabet, N.E. assessed the quantitative changes in right and left palatal rugae in the anteroposterior direction [19,21]. Comparison of quantitative changes in the anteroposterior direction of palatal rugae in various types of orthodontic treatment including extraction, non-extraction, head-gear, and functional appliance was reported by Bailey, L.J. et al. and Almeida, M.A. et al. [8,24]. However, the third rugae were found to be more stable in comparison with the first and second, which is concurrent with the previously reported literature on the stability in the morphologic pattern of the third rugae. Despite significant overall dimensional changes, observers were able to make a highly accurate (>90%) matching between the pre- and post-treatment casts using the morphological pattern of the rugae. This can be explained by the observations of the individual studies wherein the morphological pattern of the rugae was reported to be unchanged [16,19,25,28,31,32] despite the orthodontic treatment. Palatal rugae can thus be a reliable tool for personal identification by a combination of objective assessment of the morphologic pattern of palatal rugae and quantitative dimensional assessment, thereby reducing intraobserver and interobserver bias. Also, the findings of the present review must be viewed with caution, considering the moderate risk of bias in 11 of the included studies. Future studies assessing the changes in palatal rugae patterns following orthodontic treatment must focus on minimizing the confounding factors. Studies on the duration of stability of palatal rugae with changes in body weight also have to be conducted to determine the reliability of palatal rugae patterns as forensic tools.

## 5. Conclusions

Based on the results of the quantitative analysis, significant changes were noted in the lateral first rugae in the transverse bilateral direction (*p* = 0.02) and between the second and third lateral rugae of the left side in the anteroposterior direction (*p* = 0.04). Hence, identification made solely based on the dimensional parameters of landmarks of the palatine rugae may not be a reliable tool for personal identification. However, the qualitative review revealed that the observers, through visual comparison of the casts, were able to match pre- and post-orthodontic casts accurately (>90%). During the visual comparison, no objective measurements are taken into account. Thus, the matching is purely subjective and could potentially carry significant inter-observer bias. The accuracy (>90%) of matching noted in the review despite the non-significant dimensional changes could indicate the relative stability of the rugae morphology. It is also likely that there are changes in the morphology that are not perceived by the human eye, which, in the present scenario, led to increased accuracy in matching. Despite the high matching obtained from the visual comparison, it is vital to acknowledge its inherent subjectivity. Combining the dimensional quantitative measurements with morphology could potentially overcome the inter-observer bias and increase the accuracy of palatine rugae-based personal identification. Also, future studies must integrate 3D digital images of the palatal rugae (pre- and post-orthodontic treatment) with artificial intelligence (AI), as, unlike a human eye, the AI would be able to perceive even the mildest of changes and provide an objective assessment of the effect of the orthodontic treatment on the palatal rugae.

## Figures and Tables

**Table 1:** Summary of the data extracted from the included studies.

S.no	Author and Year	Place of Study	Sample Size	Age Group (Years)	Type of Orthodontic Treatment	Duration b/w Pre- and Post-Orthodontic Treatment	Methodology Used	Matching Results	Results	Conclusion
1	Shetty, D. et al., 2013 [16]	India	5025 M 25 F	15–30	_	_	1. Double-blind study 2. Casts distributed among two observers 3. Sex and rugae stability determined	90.32%	1. Consistency is found in the rugae pattern before and after orthodontic treatment (*p* < 0.0001) 2. Females have more divergent rugae compared to males (*p* < 0.05)	Rugae morphological pattern can be useful in forensic sciences compared to other body parts in cases of mutilation
2	Taneva, E.D. et al., 2015 [17]	USA	45 digital maxillary models of 15 adolescents	12–18	_	20–24 months	Cast analysis is conducted for transverse deviations with I-Tero intraoral scans (18 2D variables) and Ortho Insight 3D scanner (13 3D variables)	_	1. 18 2D variables show significant mean differences before and after treatment. (*p* = 0.001) 2. There were no statistically significant mean differences reported for 13 3D variables (*p* > 0.05) 3. Linear images are not enough to evaluate the changes in palatal rugae	Lateral and medial points of 3rd palatal rugae are suggested reliable for verifying a person’s identity
3	Kapoor, P. and Miglani, R., 2015 [18]	India	1410 F, 4 M	12 to 14	Rapid maxillary expansion	_	Cast analysis—transverse deviations including inter-medial and inter-lateral distances of the three rugae recorded with the help of Vernier calipers	_	1. Unanimous increase in inter-medial and inter-lateral distances of all rugae 2. The statistically significant increase between medial aspects of 2nd (*p* = 0.02) and 3rd rugae (*p* = 0.05) 3. Minimum increase in inter-medial distance of first rugae (0.14 mm) 4. Statistically significant increase between lateral aspects of 1st (*p* = 0.015), 2nd (*p* = 0.006), and 3rd (*p* = 0.001) rugae 5. The maximum increase in inter-lateral distance of 3rd rugae (1.42 mm) 6. Transverse changes were recorded in the order of 3rd > 2nd > 1st rugae	Medial and lateral points of 2nd and 3rd rugae cannot be used as reliable markers for forensic identification
4	Shukla, D. et al., 2011 [19]	India	50 subjects21 M 29 F	18–27	Retraction of maxillary anterior teeth with and without extraction	8–30 months	1. Cast analysis for transverse linear distances and anteroposterior distances between medial and lateral points of the three rugae were calculated 2. Morphological features affecting the accuracy of rugae identification were also evaluated	74–98%	A significant difference in transverse and anteroposterior measurements for lateral points of first rugae and between first and second rugae, respectively (*p* < 0.05)	Morphology of rugae remains unaltered and hence can be used as a reliable forensic marker
5	Jang, I. et al., 2009 [20]	Japan	10 subjects4 M 6 F	15–27	Maxillary retraction	_	1. 3D images of the cast were superimposed using the mini-screws superimposition method and Rugae–palate superimposition method 2. Cast analysis was carried out for transverse measurements	_	1. Largest displacement in lateral first rugae 2. Minimum displacement in medial third rugae hence can be used as stable landmarks 3. The difference in displacement seen in the two superimposition models is not statistically significant (*p* < 0.01)	Medial points of 3rd palatal rugae are stable enough to be used as reliable markers for forensic identification
6	Aziz HMA and Sabet NE, 2001 [21]	Egypt	50 27 F 23 M	17–25	Bilateral extraction of premolars with canine retraction	_	1. Scanning of the cast was conducted using Simplex DP 30 computer scanner 2. Transverse linear distances and anteroposterior distances between medial and lateral points of the three rugae were calculated 3. The perpendicular distance from mid-palatal raphae to medial and lateral points of rugae	_	1. Insignificant differences between lateral points of second and medial points of third rugae were observed. (*p* > 0.05) 2. Insignificant anteroposterior difference between second and third lateral rugae points (*p* > 0.05) 3. The changes in the position of palatine rugae with mid-palatine raphae were non-significant	1. Orthodontic treatment and tooth movement have a non-significant effect on the position of palatine rugae 2. Lateral third rugae points were considered the most reliable
7	Hoggan BR and Sadowsky C, 2001 [22]	USA	33 16 M 17 F	11 to 16	Extraction followed by retraction of incisors	18–46	1. Maxillary study casts and lateral cephalometric radiographs were studied 2. Maxillary central incisors and first permanent molars were traced 3. Molar and incisor movements relative to each rugae were evaluated before and after treatment	_	1. No significant differences between right and left first molars and incisors and their corresponding rugae points (*p* > 0.05)	1. The mean molar movement was the same whether measured using cephalometry or using study casts 2. The medial end of the third palatal rugae is a suitable landmark for serial model analysis of molars and incisors
8	Xiu-Ping et al., 2017 [23]	China	70 35 M 35 F	≥18	Subjected to orthodontic treatment with straight wire	12 to 24	1. Digital images of palatal rugae were taken with a DSLR camera before and after orthodontic treatment 2. Matching of these images was carried out	95.67%	The accuracy rate of matching palatal rugae patterns before and after orthodontic treatment is 95.67% with *p* < 0.05	The digital image recognition system is beneficial to human identification among large-sized samples
9	Bailey, L.J. et al., 1996 [8]	USA	57 subjects (Extraction—27; Non-extraction—30)	18–36	Extraction of maxillary premolars followed by retraction of incisors and the non-extraction group treated by edgewise technique	_	1. Landmarks on each cast were digitized 2. Changes from initial to final records were calculated 3. Transverse offset, transverse linear, and anteroposterior measurements were taken	_	1. Significant changes were observed for transverse offset and transverse linear distances between the medial points of first rugae in the non-extraction group and lateral points of first and second rugae in the extraction group 2. Anteroposteriorly significant changes were observed between medial and lateral points of all the rugae in the extraction group only	Medial and lateral points of third palatine rugae were considered stable landmarks in transverse, linear and anteroposterior direction irrespective of the treatment conducted with or without extraction
10	Almeida, M.A. et al., 1995 [24]	USA	94 childrenCntrl 34 HG 30 FC 30	_	Orthodontic treatment for early class II cases with headgear and functional appliance	0–15	1. Landmarks were made on mid-palatal raphae and palatine rugae 2. Perpendicular distances from MPP to rugae3. Transverse and anteroposterior distances between medial and lateral points of palatine rugae	_	1. Lateral rugae offset changes were significantly different among the three groups (*p* < 0.01) 2. Medial points of first rugae were stable regardless of the treatment (*p* > 0.01) 3. Anteroposteriorly only head-gear group showed an increase between medial points of rugae	Medial rugae points can be used as stable reference landmarks for longitudinal cast analysis in transverse and anteroposterior planes
11	Li, B. et al., 2017 [25]	China	70 35 F 35 M	≥18 years	Orthodontic treatment with straight wire	12 to 24	1. Matching test of rugae was performed by 10 dentists	99.05%	1. Distal endpoint displacement anteroposteriorly and mesiodistally in 45.7% and 40% of patients, respectively 2. Mesial endpoint displacement anteroposteriorly and mesiodistally in 32.9% and 17.1% of patients, respectively	1. Palatal rugae show diverse presentation patterns after orthodontic treatment 2. Still, they can be used as a reliable marker in forensic identification
12	Stavrianos, C. et al., 2012 [26]	Greece	50	≥14 years	_	18 months to 4 years	1. 50 post-orthodontic casts were mixed with 100 randomly selected casts 2. 5 evaluators were asked to match 50 pre-orthodontic casts with those of 50 post-orthodontic ones	94–100%	1. Each set of pre- and post-orthodontic casts were 100% correctly matched by 4 evaluators 2. Owing to less experience in the field, the fifth one could manage to match it by 94% (*p* = 0.05)	Palatal rugae can be used for human identification by comparing ante- and post-mortem data
13	Samyukta S and Abhlasha R, 2016 [27]	India	20 10 M 10 F	_	_	_	1. Intercanine distance was measured between cusp-tips 2. Medial and lateral points of third rugae were marked3. The ratio between inter-canine width and lateral dimension of third rugae was evaluated	_	1. Mean value of the ratio was not significantly different in pre- or post-treatment casts (*p* < 0.05) 2. No change in the pattern of 3rd rugae in both sexes	Palatine rugae can be used as stable markers for personal identification
14	Braga S and Caldas IM, 2015 [28]	Portugal	4624 F 22 M	22–31 years	_	_	1. Dental casts were photographed using a digital camera 2. All three rugae on both sides were analyzed	_	1. Males—no significant difference between rugae length before and after treatment (*p* > 0.05) 2. Females—differences between first right rugae initial and final lengths were reported (*p* = 0.039) 3. No significant differences were found in morphology (*p* > 0.05)	Palatal rugae morphology is a reliable marker for human identification
15	Deepak, V. et al., 2015 [29]	India	137(extraction, 50; non-extraction, 50; palatal expansion, 37	_	Orthodontic treatment was carried out by pre-adjusted edgewise therapy	8–24 months	Rugae length, shapes, and positions were recorded on both right and left sides of pre- and post-orthodontically treated casts	_	1. Right—not much difference was observed in length in the extraction group, while there was an increase in length in non-extraction and palatal expansion 2. Left—no significant difference in non-extraction and palatal expansion but a slight increase in length in the extraction group 3. Maximum changes were seen in the palatal expansion and extraction group and minimum changes in the non-extraction group on both right and left sides (*p* = 0.00041)	1. Most stable reliable points are of 3rd rugae 2. Role of palatal rugae in individual identification remains questionable
16	Mustafa, A.G. et al., 2015 [30]	Jordan	50 pairs of casts	_	_	_	Casts were assessed for palatine rugae for their change in number, orientation, shape, length, and displacement of medial and lateral ends mediolaterally and anteroposteriorly	90–99% (*p* < 0.05)	Change in number—20–22% Change in orientation—6% Change in shape—6% Change in length—28% Antero-posterior displacement—60% Medio-lateral displacement—28%	The orthodontic treatment induces morphometric changes in palatal rugae which complicate rugae based human identification
17	Ali, B. et al., 2016 [31]	Pakistan	56 subjects28 M 28 F	_	Subjects were treated with fixed orthodontic appliances with or without extraction and with RPE	28–33 months	1. Dental casts were divided into 3 groups including extraction, non-extraction, and palatal expansion 2. Casts were determined at two-time intervals for lengths and shapes	_	1. Extraction—lengths of 2nd and 3rd rugae are significantly reduced (*p* < 0.05) 2. Palatal expansion—3rd rugae length was significantly increased (*p* < 0.05) 3. No significant change in shapes of rugae was reported (*p* > 0.05)	The shape of the rugae can be used as a reliable marker for individual identification in forensic sciences
18	Shailaja, A.M. et al., 2018 [32]	India	15 subjects7 M 8 F	10–13 years	The fixed rapid maxillary expansion was used to correct anterior or posterior crossbite in all the cases	_	1. Transverse distance between medial and lateral points of the first two rugae and last two rugae have been evaluated 2. Inter-medial and inter-lateral distance have also been assessed 3. Shapes of the rugae were also assessed	_	1. A statistically significant difference in the distance between medial and lateral points of 1–2 and 2–3 rugae (*p* < 0.0001) 2. A statistically significant difference in the transverse distance between medial and lateral points of rugae (*p* < 0.0001)	Morphological pattern of rugae can be used as a reliable marker in forensic identification

**Table 2:** Joanna Brigg’s risk of bias assessment of the included studies.

First authors Names/Year of Publication	Were the Criteria for Inclusion in the Sample Clearly Defined?	Were the Study Subjects and the Setting Described in Detail?	Was the Exposure (Treatment) Measured Validly and Reliably?	Were Objective, Standard Criteria Used for Measurement of the Condition?	Were the Confounding Factors Identified?	Were Strategies to Deal with Confounding Factors Stated?	Were the Outcomes Measured Validly and Reliably?	Was an Appropriate Statistical Analysis Used?	The Overall Risk of Bias
Shetty, D. et al., 2013	No	Yes	Yes	Yes	Yes	Yes	Yes	Yes	Low
Taneva, E.D. et al., 2015	Yes	Yes	Yes	Yes	Unclear	No	Yes	Yes	Low
Kapoor, P. and Miglani, R., 2015	Yes	Yes	Yes	Yes	No	No	Yes	Yes	Low
Shukla, D. et al., 2011	No	Yes	Yes	Yes	No	No	Yes	Yes	Moderate
Jang, I. et al., 2009	No	Yes	Yes	Yes	No	No	Yes	Yes	Moderate
Aziz, H.M.A. and Sabet, N.E., 2001	Yes	Yes	Yes	Yes	No	No	Yes	Yes	Low
Hoggan, B.R. and Sadowsky, C., 2001	No	Yes	Yes	Yes	No	No	Yes	Yes	Moderate
Xiu-Ping, W. et al., 2017	Yes	Yes	Yes	Yes	Yes	Yes	Yes	Yes	Low
Bailey, L.J. et al., 1996	No	Yes	Yes	Yes	No	No	Yes	Yes	Moderate
Almeida, M.A. et al., 1995	No	Yes	Yes	Yes	No	No	Yes	Yes	Moderate
Bing, L. et al., 2017	Yes	Yes	Yes	Yes	Yes	Yes	Yes	Yes	Low
Stavrianos, C. et al., 2012	No	Yes	Yes	Yes	No	No	Yes	Yes	Moderate
Samyukta, S. & Abhlasha, R., 2016	No	No	Yes	Yes	No	No	Yes	Yes	Moderate
Braga, S. & Caldas, I.M., 2015	Yes	Yes	Yes	Yes	No	No	Yes	Yes	Low
Deepak, V. et al., 2015	No	No	Yes	Yes	No	No	Yes	Yes	Moderate
Mustafa, A.G. et al., 2015	No	No	Yes	Yes	No	No	Yes	Yes	Moderate
Ali, B. et al., 2016	No	No	Yes	Yes	No	No	Yes	Yes	Moderate
Shailaja, A.M. et al., 2018	Yes	No	Yes	Yes	No	No	Yes	Yes	Moderate

## Data Availability

Not applicable.
